# The importance of measuring the levels of ionized magnesium in the blood in critically ill patients

**DOI:** 10.5937/jomb0-54782

**Published:** 2025-06-13

**Authors:** Marija B. Stanojević, Miro Parezanovic, Marko Biorac, Svetolik Spasić, Sanjin Kovačević, Srđan Lopičić, Ostojić Jelena Nešović

**Affiliations:** 1 University of Belgrade, Faculty of Medicine, Institute for Pathological Physiology "Ljubodrag Buba Mihailović", Belgrade; 2 Institute for Mother and Child Healthcare of Serbia "Dr Vukan Čupić", Belgrade

**Keywords:** magnesium deficiency, critically ill, ionized magnesium, magnesium measuring, deficit magnezijuma, jonizovani magnezijum, kritično oboleli pacijenti, merenje magnezijuma

## Abstract

**Background:**

Critical illnesses imply vital organ dysfunctions with high risk of imminent death. Magnesium is a bioessential element with multiple physiological effects relevant for vital body functions. It stabilizes excitable membranes contributing to proper neuromuscular, cardiovascular and respiratory functions. Ionized Mg2+ (iMg2+) is free bioactive form of Mg2+ in body fluids. Magnesium disorders in critically ill patients are often overlooked. Chronic Mg2+ deficiency is a condition of growing incidence in the general population and a significant factor in overall morbidity and mortality in critical illness. Main goal of this study was to investigate the existing literature related to determination of iMg2+ in the critically ill and to raise awareness of the problem of chronic Mg2+ deficiency in these patients.

**Methods:**

Search was conducted across electronic PubMed library database from 1975 to November 2024 using keywords concerning iMg2+ and critical care patients, to identify studies investigating the measuring of blood concentration of iMg2+ fraction in patients with critical health conditions. Search was limited to English language. Selection criteria included only studies on human, and excluded studies on animal population.

**Results:**

We identified 95 relevant studies. Reviewed papers show that abnormalities of Mg2+ levels are prevalent in critical illnesses. Their severity can impose vital threat. Disconcordance between total Mg2+ (tMg2+) and iMg2+ blood levels is frequently present in critical patients.

**Conclusions:**

Appropriate electrolyte analyzer assay is needed to promptly determine iMg2+ levels to assess Mg2+ status in critically ill patients, in order to help detect and correct Mg2+ imbalances and estimate the requirement for Mg2+ recompense in the case of deficiency, and thereby provide better outcome of the disease.

## Abstract (sr)

**Uvod:**

Kritične bolesti podrazumevaju disfunkciju vitalnih organa sa visokim rizikom od prete}eg smrtnog ishoda. Magnezijum je bioesencijalni element sa višestrukim fiziološkim dejstvima značajnim za vitalne funkcije organizma. Magnezijum stabilizuje ekscitabilne membrane, doprinoseći pravilnoj neuromišićnoj, kardiovaskulnoj i respiratornoj funkciji. Jonizovani Mg2+ (iMg2+) je slobodni bioaktivni oblik Mg2+ u telesnim tečnostima. Poremećaji Mg2+ statusa kod kritično obolelih pacijenata često se zanemaruju. Hronični deficit Mg2+ je stanje rastuće učestalosti u opštoj populaciji i predstavlja značajan faktor ukupnog morbiditeta i mortaliteta kritično obolelih. Osnovni cilj ovog rada je bio da se ispita postojeća literatura u vezi sa određivanjem koncentracije iMg2+ kod kritično obolelih i da se podigne svest o problemu hroničnog deficita Mg2+ kod ovih pacijenata.

**Metode:**

Sprovedena je elektronska pretraga bibliotečke baze podataka PubMed od 1975. do novembra 2024. godine, koristeći ključne reči koje se odnose na iMg2+ i kritično obolele pacijente, da bi se identifikovale studije koje su merile koncentraciju frakcije iMg2+ u krvi kod pacijenata sa kritičnim poremećajima zdravlja. Pretraga je bila ograničena na engleski jezik. Kriterijumi za odabir uključivali su samo studije na ljudima i isključivali studije na životinjama.

**Rezultati:**

Identifikovali smo 95 studija od značaja. Pregledani radovi pokazuju da su abnormalnosti nivoa Mg2+ veoma česte u kritičnim bolestima. Teži poremećaji mogu dovesti i do vitalne ugroženosti. Neslaganje izme|u nivoa ukupnog Mg2+ (tMg2+) i iMg2+ u krvi često je prisutno kod kritično obolelih.

**Zaključak:**

U kritično obolelih pacijenata je neophodno određivanje nivoa iMg2+ u krvi odgovarajućim elektrolitnim analizerom radi brze procene Mg2+ statusa, otkrivanja i korigovanja poremećaja ravnoteže Mg2+, kao i procene potrebe za nadoknadom Mg2+ u slučaju njegovog nedostatka, a u cilju da se obezbedi bolja prognoza ishoda bolesti.

## Introduction

Critical care of hospitalized patients is needed at surgery departments, emergency departments (EDs) and intensive care units (ICUs). Critical care medicine requires diagnostic procedures performed with critical care portable or transportable instruments at or near patient’s bedside which can eficiently guide patient management and treatment. An important advance in this field are key biochemical test with results quickly available at point-of-care to the attending clinician. Managing critically ill, critically injured and vitally endangered patients among other things includes monitoring blood electrolytes in order to assess the status of water and salt metabolism and detect its disorders if present in the body, that can seriously influence patient morbidity and mortality. Electrolytes in the blood are obligatory analytes in the profile of critical care tests. Within a brief period of time these results can be used to effectively guide stabilization of vital body functions and general health condition of the critical patient [1]. As an electrolyte, magnesium (Mg^2+^) is the fourth most abundant cation in the body. However, clinical utility of measurable Mg^2+^ is still underused. Magnesium is unfortunately frequently absent from the routine plasma or serum ionogram panels, and its importance for general human health is still neglected.

Maintaining Mg^2+^ homeostasis in the body is important for preserving numerous biological processes, including vital body functions. Magnesium is a bioessential mineral that plays numerous roles in a number of processes important for living organisms on all levels. On a subcellular and cellular level, Mg^2+^ ion participates in the regulation of ion fluxes of other cations (H^+^, Na^+^, K^+^ and Ca^2+^) through biological membranes [2]. This includes Mg^2+^ regulation of function of ion pumps and other ion transport mechanisms, oxidative phosphorylation and cell energy meta bolism, as well as the processes of cell excitability and conductivity and synaptic neurotransmission. A stable concentration ratio for major cations Na+ : K^+^ : Ca^2+^ : Mg^2+^ is preserved under physiological conditions (cation isoiony), to help maintain normal level of neuromuscular excitability. On tissue, organ, organ systems level and eventually the level of organism as a whole, Mg^2+^ participates in the regulation of smooth muscle tone, skeletal muscles and heart muscle contractility, arterial blood pressure and pulmo nary blood flow, glycaemia and insulin levels, protection against oxidative stress and chronic inflam mation, and more.

Magnesium deficiency is an electrolyte disturbance due to a low level of Mg^2+^ in the body and adiagnostic challenge. It is a much more prevalent type of disorder of Mg^2+^ homeostasis than Mg^2+^ excess. Namely, there is a significant decrease in dietary magnesium intake over the past century. Magnesiumpoor foodstuff and processed food are becoming common in the diet of everyday life. Besides the inadequately low intake, other causes can also contribute to the condition of chronic Mg^2+^ deficiency (gastrointestinal issues, increased renal wasting, intracellular redistribution etc.). Alcohol consumption increases magnesiuresis, adding to the negative Mg^2+^ balance. This subtle Mg^2+^ deficiency is nowadays present in a large number of apparently ”normal” individuals, representing an issue of rising medical impact in the general population. Chronic Mg^2+^ deficiency can remain asymptomatic, i.e. clinically latent by releasing Mg^2+^ from body pools (especially skeletal muscle and bone Mg^2+^), maintaining stable Mg^2+^ levels in the blood for a long time. These individuals would actually need adjustment of their diet or supplementation with magnesium to achieve a normal Mg^2+^ status for health, and preserve adequate bone density to prevent osteoporosis. Because of the tendency of chronic subclinical Mg^2+^ deficiency to become even more prevalent, some authors believe that concerning the concentration of total Mg^2+^ (tMg^2+^) in the blood – a new appropriate lower limit of the reference interval for health should be established, and recommend increasing it to 0.85 mmol/L Mg^2+^ [3].

Chronic latent Mg^2+^ deficiency is considered to be especially prevalent in the developed and developing countries and it occurs more often in the elderly then in the young ones [4]. Because Mg^2+^ has an important role in the maintenance of stability of excitable membranes and protection against oxidative stress and chronic inflammation, chronic Mg^2+^ depletion is assumed to contribute to the pathogenesis of a number of pathophysiological conditions and chronic non-communicable diseases (cardiac arrhythmias, atherosclerosis, arterial and pulmonary hypertension, coronary heart disease, certain forms of heart failure, bronchoconstriction, epilepsy, migraine, tension headache, neurodegenerative conditions, anxiety, depression, chronic pain, etc.). One of the underlying mechanisms can be an overall change in general function of excitable tissues and organs in a form of low level hyperexcitability that develops and chronically persists in the state of chronic Mg^2+^ deficiency [5].

Electrolyte disorders are common in hospitalized patients. Critical care patients are very susceptible to alterations in electrolyte balance. Acute and chronic disorders of Mg^2+^ balance are also among common electrolyte abnormalities in the critically ill. Surgical patients frequently develop acute Mg^2+^ deficiency with a significant intraoperative and postoperative depletion of Mg^2+^, due to stress [6]. However, despite all these findings, there is a big gap between basic Mg^2+^ research and clinical practicioners dealing with Mg^2+^ disorders. Although the importance of Mg^2+^ is scientifically well recognized, too little attention is paid to its disbalances in clinical practice. Magnesium is still not a regularly monitored electrolyte in ICU settings [7]. Given that the role of Mg^2+^ homeostasis disorders is clinically still often neglected, while Mg^2+^ deficiency in the body is rarely tested for, we believe that it would be of great benefit to introduce into clinical practice a routine monitoring of Mg^2+^ status in patients at ICUs, EDs and perioperatively, through the measurement of concentrations of both tMg^2+^ and the fraction of ionized Mg^2+^ (iMg^2+^) in the blood. This would alleviate timely detection of the latent Mg^2+^ deficiency, as a relevant risk factor for critical diseases.

### Aim

The authors generally aim to draw the attention of clinicians, especially those working at ICUs or EDs treating critically endangered patients, to the importance of testing Mg^2+^ status of the patient by measuring not only tMg^2+^ concentration, but also (and even primarily) the concentration of iMg^2+^ fraction in the blood, since iMg^2+^ is a more specific marker of Mg^2+^ status of the body.

Present literature does not show uniformity in abbreviations used for the forms of circulating Mg^2+^ (entire Mg^2+^ in the blood and its fractions). We believe that a generally accepted consensus on abbreviations for the ionized fraction of Mg^2+^ and total Mg^2+^ is needed. However, we consider that they should certainly include Mg^2+^ (with a symbol of electrical charge included), since this is one of the major electrolytes in our body fluids. From a clinical point of view, it is very important to stress the electrophysiological implications of Mg^2+^ status alterations. Pathophysiology of disorders of Mg^2+^ balance includes inevitable effects on excitable tissues and organs [5]. Concerning that most of major vital body functions require regular neuromuscular excitability that Mg^2+^ affects, we believe it is important to rise awareness of clinical practitioners, especially those dealing with critical conditions, of the electropathophysiological consequences of Mg^2+^ disorders onto vital body functions. This specific aim explains why the authors decide to preserve the symbol of ion charge in the superscript (^2+^ for Mg) wherever this electrolyte is mentioned throughout the text, even in terms of an analyte, although this is not in line with the guidelines of the International Federation of Clinical Chemistry and Laboratory Medicine for ionized magnesium (which suggest only iMg as an abbreviation, [8]). Similarly, the very term »ionized« should also be additionally explained. Here it can actually be considered a misnomer, given that all of Mg^2+^ in the body water is in ionized or ionic (and not atomic) form. However, we decided to keep using this already popularized term to refer to the free form of Mg^2+^, unbound to any anion.

## Materials and methods

We searched the PubMed library electronic database from 1975 to November 30, 2024 using combinations of the following keywords: ionized magnesium AND *critical care* OR *critical patients* OR *critically ill*. Literature search was limited to scientific publications with full text in English language. Articles from reference lists included clinical studies (prospective and retrospective, observational studies and randomised controlled clinical trials etc.) and review papers. Only studies and reviews concerning human population were included, while studies on animals were excluded from the search. Critical conditions from different medical fields were included (coronary units, cardiopulmonary surgery, neurocritical care, surgical intensive care, liver diseases, renal diseases, obstetrics etc.), both in adults and children. Blood samples for determination of Mg^2+^ levels included whole blood, blood plasma and blood serum. Applied methods for the measurement of Mg^2+^ levels mostly included ion-selective electrodes, atomic absorption spectrometry and dye binding method. After screening the literature, articles of interest were retrieved and examined. Subsequently additional papers of potential relevance were included.

This paper does not deal with preanalytical and analytical issues and problems concerning sampling, measuring and reporting iMg^2+^ in different blood samples, but rather with pathophysiological and clinical aspects of determination and monitoring of iMg^2+^ concentrations in critically ill patients in different clinical conditions. A review of the literature was made on the importance of monitoring the concentration of iMg^2+^ fraction in the blood of critical care patients, to raise awareness of the problem of its insufficient clinical determination. Additionally, chronic latent Mg^2+^ deficiency in the body is discussed to be an independent risk factor for the onset and maintenance of many common chronic non-communicable diseases, including those leading to life-threatening conditions that endanger critically ill patients. The role of measuring iMg^2+^ levels in discovering chronic latent Mg^2+^ deficiency is explained.

## Results

A total of 95 relevant articles were identified according to defined search criteria over a 50-years long time period. A relatively small number of studies found in this literature search is a witness of insufficient clinical concern for the questions of measuring iMg^2+^ and assessing Mg^2+^ status in the critically ill, and/or objective obstacles to reliably perform such measurements. A misconception that normal tMg^2+^ levels exclude disorders of Mg^2+^ balance probably contributes significantly. One of the objective reasons is insufficient availability of required specific electrolyte analyzers at ICU departments due to high costs. Another potential reason is a neglect of Mg^2+^ as an electrolyte, seen in clinical practice more or less in general and not restricted only to critical care [3]. It seems that physicians do not relate adequately iMg^2+^ levels and Mg^2+^ status on one hand, with vital body functions and critical conditions on another. For comparison, when we performed the same article search under the same criteria as for *ionized magnesium*, only using *ionized calcium* as a keyword, the number of articles found was four times bigger than that for magnesium. This illustrates the amount of neglect of Mg^2+^ as compared to Ca^2+^ regarding critical care patients. Infrequent detection of iMg^2+^ in clinical practice can partially be due to a lack of standardization of diagnostic reference ranges [9].

We summarized search results we found into several chapters of this review to analyze the following selected questions:

- Why is it clinically significant to measure Mg^2+^ concentrations in the blood?
- What total magnesaemia and ionized magnesaemia represent?
- Can iMg^2+^ be used as an indicator of Mg^2+^ status of the body?
- How to interpret the results of measuring Mg^2+^ concentrations in the blood?
- Why is it important to think of chronic Mg^2+^ deficiency in the critically ill?
- How to guide the correction of disorders of Mg^2+^ status in critically ill patients using iMg^2+^?

Answers to the questions raised are presented by specific results of the previously published works identified in literature search, and discussed by the authors.

### Clinical significance of measuring Mg^2+^ concentration in the blood

Measuring the concentration of individual electrolytes in the blood helps determine electrolyte status in the blood, which reflects the state of general electrolyte metabolism in the body. Blood electrolyte findings are informative and easily available, however, they are of limited clinical value when it comes to monitoring the overall body status of certain ions, namely the dominantly intracellular ones. Distur - bances of Mg^2+^ homeostasis can lead to dysregulation of Mg^2+^ levels in the blood. Dys magnesaemia implies a disorder of tMg^2+^ concentration in the blood and occurs with a variable frequency estimated to be about 2% in the general population, 10–20% in the hospitalized patients, 50–60% of patients at ICUs and up to 30–80% of alcoholics [10]. Highly prevalent dysmagnesaemia in the hospitalized patients is shown to have prognostic value as a predictor of poor patient outcome. Abnormal Mg^2+^ levels are also very common in children admitted to pediatric ICUs and associated with higher mortality [11].

The ICU and ED physicians should be aware of clinical conditions that predispose to severe dysregulations of Mg^2+^ metabolism, as these patients develop profound cardiovascular, respiratory, neuromuscular and neurological dysfunctions [12], due to a significant effect of Mg^2+^ disbalances on the physiology of excitable membranes and their strong impact on a number of vitally important body functions. This mainly results from Mg^2+^ being active on excitable membranes, which is known for quite some time from basic electrophysiology. For example, a number of studies since late 1960s have described Mg^2+^–induced depression of membrane excitability in central neurons [13] [14]. Consequently, a condition of Mg^2+^ deficiency would disturb cation isoiony, leading to a generalized state of membrane hyperexcitability. Unfortunately, it seams like these preclinical findings and knowledge have never been fully implemented clinically. Similarly, the necessity to include Mg^2+^ into the laboratory diagnostics at the point-of-care is not fully appreciated yet. However, disorders of Mg^2+^ homeostasis can directly affect heart action, blood flow, breathing, neuromuscular excitability and conductivity, and metabolism regulation [15]. Therefore, any dysmagnesaemia (both hypo- and hypermagnesaemia) is especially dangerous for the critically ill patient, implying its importance for the fields of emergency medicine and intensivecare. Table 1 summarizes the most important disorders of vital functions that dysmagnesaemias cause or contribute to.

**Table 1:** Dysmagnesaemias – clinical manifestations.

**Hypomagnesaemia**	arterial hypertension, tachyarrhythmias, coronary vasospasm, development of heart<br>failure, epileptic seizures, hyperreflexia, skeletal muscle weakness (including respiratory<br>muscles), tetany, depression, migraine, hypokalaemia, hypocalcaemia
**Hypermagnesaemia**	arterial hypotension, bradycardia, heart blocks, cardiac arrest, respiratory depression,<br>muscle paralysis, confusion, lethargy, hyporeflexia - areflexia, decreased platelet aggregation<br>and blood clotting

Magnesium disorders can exist before or develop after admission and progress during hospitalization. For example, therapeutic plasma exchange in acute liver failure patients induces a significant fall in Mg^2+^ concentrations in the blood and a profound ionized hypomagnesaemia [16]. Similarly, hypomagnesaemia is a common electrolyte disorder in patients with acute kidney injury, in whom relevant additional electrolyte losses via the filter may occur during treatment. Using renal replacement therapy with citrate anticoagulation or inadequate dialysis solutions can further worsen the existing hypomagnesaemia, which imposes the need to closely monitor Mg^2+^ levels in these patients [17].

### Total magnesaemia vs. ionized magnesaemia

**Figure 1:**
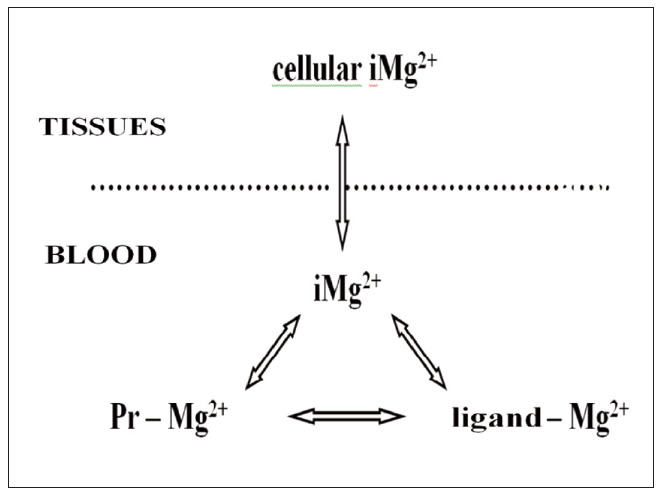
The largest proportion of the total amount of Mg^2+^ present in the blood is in the form of free Mg^2+^ ions (iMg^2+^ : ionized Mg^2+^). The remaining part consists of bound Mg^2+^ (ligand – Mg^2+^ : Mg^2+^ complexed with anionic ligands, and Pr – Mg^2+^: Mg^2+^ bound to plasma proteins). Only free iMg^2+^ is bioactive, while bound Mg^2+^ is not available for biological processes. Binding of Mg^2+^ in the plasma depends on the presence of plasma proteins, other ligands and plasma pH value, especially important in critical care patients. Extracellular iMg^2+^ is in a dynamic equilibrium with cellular and tissue iMg^2+^. Pr – proteins

In all our body fluids, Mg^2+^ exists in equilibrium between free form and bound forms of Mg^2+^. Total Mg^2+^ in the blood occurs in three forms: iMg^2+^ (59–72%), Mg^2+^ bound to proteins (23–31%) and Mg^2+^ bound in complexes with various ligands (5–11%) [18]. Free Mg^2+^ ions are being continuously dynamically interchanged between the three Mg^2+^ fractions in the blood: ionized, protein-bound and ligandbound complexed Mg^2+^. Figure 1 shows the dynamic balance between Mg^2+^ fractions in the blood.

Fractions of Mg^2+^ bound to proteins (mainly albumins) and Mg^2+^ complexed with other anions (sulfates, phosphates, bicarbonates, lactates, acetates, citrates, oxalates, amino acids, free fatty acids, etc.) together comprise bound Mg^2+^. Bound Mg^2+^ contributes to the total pool of circulating Mg^2+^ and represents a form of Mg^2+^ reservoir in the blood. However, bound Mg^2+^ is biologically inert, as it is not free to contribute to the many physiological effects of Mg^2+^. Thereby, tMg^2+^ level which is usually measured due to the lack of alternatives, has only limited informative value. Only free iMg^2+^ is the active fraction of the circulating Mg^2+^, always available for biological effects and easily exchangeable with intracellular Mg^2+^. Hence, the main Mg^2+^ fraction in the state of preserved Mg^2+^ homeostasis and adequate Mg^2+^ status of the organism is actually the iMg^2+^.

Concerning reference ranges for Mg^2+^ concentrations, values depend on the sample used, themeasurement method applied and the type of analyzer. Values can also vary depending on the age and sex of the examinee. On average, Mg^2+^ levels are physiologically always slightly higher in adults than children, and in men than in women across all age groups [19]. However, current literature does not show uniform reference ranges for tMg^2+^ and iMg^2+^ levels. Some authors believe that defined equations containing some routinely assessed serum parameters (like tMg^2+^ levels, Ca^2+^ levels and albumin) and demographic variables can be used to accurately estimate serum iMg^2+^ levels, in order to enable guided medical treatment and improved patient care by the treating physicians [18]. Others state that iMg^2+^ is difficult to calculate and suggest that it should be directly measured in order to increase the reliability of determination [20]. We also consider that a reliable finding of iMg^2+^ levels can only be obtained by direct measurements and not by calculations. Reference ranges for Mg^2+^ levels can vary depending on the type of analyzer and should be given by the manufacturer. General reference intervals for healthy adults are shown in Table 2 [21].

**Table 2:** Concentrations of total Mg^2+^ (tMg^2+^) and ionized Mg^2+^ (iMg^2+^) in the serum of healthy adult subjects – reference values.

**total magnesaemia (tMg^2+^)**<br>[mmol/L]	0.66–1.07
**ionized magnesaemia (iMg2+)**<br>[mmol/L]	0.43–0.54

Findings on Mg^2+^ status in the blood are influenced by changes in the level of hydration and circulating volume of a patient, as well as changes in plasma protein homeostasis, since disorders of proteinaemia directly change the level of Mg^2+^ fraction bound to plasma proteins. Therefore, changes in protein-bound Mg^2+^ level in conditions of hypoproteinaemia or hyperproteinaemia lower or increase tMg^2+^ respectively, without influencing the level of free iMg2+ in the blood. Since concentration of iMg^2+^ in the blood can also vary significantly under various pathophysiological conditions, it is important to measure iMg^2+^ separately. It is greatly influenced by other homeostasis, such as the acid-base balance. The acidity of the environment greatly affects the dynamic balance between the free iMg^2+^ fraction and the albumin-bound Mg^2+^ fraction (due to a competition of H^+^ and Mg^2+^ for the same ion-binding sites on plasma protein molecules). Therefore, alterations of the acid-base balance affect Mg2+ status, as they instantly and directly change iMg^2+^ levels, by changing the levels of protein-bound Mg^2+^ fraction in the opposite direction. Namely, acid-base disorders cause redistribution between iMg^2+^ and protein-bound Mg^2+^ in the blood, so that acidosis increases, whereas alkalosis decreases iMg^2+^ blood levels, while tMg^2+^ levels remain unchanged.

Magnesium derangements are also common in critically ill children, both hypo- and hypermagnesaemia. In pediatric patients admitted to a pediatric ICU, abnormal serum levels of iMg^2+^ are found more often than abnormal serum levels of ionized Ca^2+^ [22]. In patients admitted to ICU, monitoring iMg^2+^ can improve chances for a favourable outcome, as it helps discover Mg^2+^ disturbances associated with adverse ICU outcomes, better that tMg^2+^ [18] [23]. Unfortunately, conventional laboratory measurements from blood samples of the examined subjects, including ICU patients, frequently determine only tMg^2+^ concentration, not distinguishing between different Mg^2+^ fractions in the blood. Thereby, concerning Mg^2+^ levels in critically ill patients, clinical practice in general is usually still limited to using only total magnesaemia as the most common laboratory assessment of Mg^2+^ status, while ionized magnesaemia is regarded as an alternative or optional parameter, or it is not taken into consideration. It seems that the low sensitivity of tMg^2+^ levels as a biomarker of Mg^2+^ status is probably still little known among the physicians [24]. Some authors suggest that iMg^2+^ : tMg^2+^ ratio may be a valuable prognostic parameter that should be investigated in future large-scale studies [25].

### Ionized Mg^2+^ as an indicator of Mg^2+^ status of the body

Blood is the most available sample of the extracellular fluid which can readily be taken for laboratory analysis. Although only a very small amount of body’s total amount of Mg^2+^ is found in the blood, assessing Mg^2+^ status in the blood can be clinically used to evaluate oneʼs general Mg^2+^ status. Heparinized whole blood or blood plasma are preferred specimens for Mg^2+^ blood tests, since using other anticoagulants (citrate, oxalate, EDTA) renders false low levels, as they bind Mg^2+^ into stable complexes. The observation of Mg^2+^ deficiency in all examenees, and specifically in critically ill patients should be obtained only by appropriate direct measurements. This especially refers to the iMg^2+^ levels, as the active Mg^2+^ portion. Protein-bound Mg^2+^ and ligand-bound Mg^2+^ are not physiologically active, hence, tMg^2+^ in the blood does not reflect active Mg^2+^ status. Rather the level of iMg^2+^ should be tested for when diagnosing Mg2+ deficiency. Besides, free iMg^2+^ is in a dynamic equilibrium in between all the body fluid compartments, so that the concentration of iMg^2+^ in the blood is approximately the same as the concentration of free (ionized) Mg^2+^ in the cells [5]. This further confirms the statement that iMg^2+^ is generally a better indicator of Mg^2+^ balance in the tissues and body as a whole, than tMg^2+^.

Although current ion-selective electrode technology can be used to measure the concentration of iMg^2+^ in routine clinical practice, issues arise that may alter the results of measuring, concerning sample choice (whole blood, blood plasma or blood serum) and sample handling, sample container material etc. [26]. There is a need to standardize the measuring procedure in a way that would interfere with iMg^2+^ determination in the slightest possible degree. In spite of the difficulties in measuring, the level of iMg^2+^ in the blood is still considered to be the preferred clinical biomarker of Mg^2+^ status that can help identify critical care patients at risk of adverse outcomes [9]. Ionized Mg^2+^ is actually being considered as a promising new parameter for point-of-care laboratory diagnostics and optimal support of critical care patients for several decades already [27]. Its concentration in the blood can easily be measured and monitored in clinical, even urgent settings, to prevent the development of serious and potentially fatal complications in critically ill patients [28]. However, even today there is a certain undefined yet strong practical inertia that still prevents realization of this idea in daily clinical practice, probably mostly due to a limited availability of the measuring instruments at departments. Zaloga et al. [29] state that ultrafilterable Mg^2+^ values approximate iMg^2+^ levels and offer an alternative to iMg^2+^ measurement when an ion-selective electrode is not available.

Magnesium deficiency in the body is generally a clinically under-recognized electrolyte imbalance, particularly in critically ill patients, adults and children [19]. For example, a significantly lower iMg^2+^ level is found in critically ill children as compared to healthy children [30], although additional independent clinical studies are needed on the role of disturbances in iMg^2+^ in critically ill neonatal and pediatric patients [31]. In patients with acute myocardial infarction in the first days after the onset of acute chest pain simultaneous monitoring of both tMg^2+^ and iMg^2+^ found stable tMg^2+^ levels and reduced iMg^2+^ levels, whereby monitoring iMg^2+^ proved to be clinically rather more useful than monitoring only tMg^2+^ in these patients [32]. Furthurmore, in ICU patients levels of iMg^2+^ and tMg^2+^ were found not to correlate well in about 20% of cases. It is possible that an ICU patient has e.g. a finding of normal iMg^2+^ with a reduced tMg^2+^, or a finding of an increased iMg^2+^ with a normal tMg^2+^ (in both of these cases actually the level of bound Mg^2+^ is reduced, sometimes due to hypoproteinaemia). For example, in severe liver disease and dysfunction, correction of Mg^2+^ status guided by tMg^2+^ levels may lead to patient overdose, due to present hypoalbuminemia [23]. For such patients, therapy given only based on tMg^2+^ levels would actuallybe inapropriate, because it could iatrogenically induce hypermagnesaemia [20].

### Interpretating the results of measuring Mg^2+^ concentrations in the blood

Concluding on what Mg^2+^ status of a patient is only according to the value of tMg^2+^ levels is not only very challenging, but can be quite erroneous. For example, by measuring the concentrations of iMg^2+^ and tMg^2+^ in patients at surgical ICUs, it was concluded that monitoring only tMg^2+^ in patientʼs blood in a large number of cases can lead to making a medical error due to a prescription of a wrong therapy that can even harm the patient, since it relies on an unreliable decision-making based on a false image of Mg^2+^ status [33]. Total magnesaemia does not always accurately detect true Mg^2+^ status. Therefore, tMg^2+^ as an isolated finding is insufficient to enable adequate conclusion on what Mg^2+^ homeostasis of the subject is. Johansson and Whiss [34] who found a week correlation of iMg^2+^ and tMg^2+^ serum levels in critically ill patients, conclude that measuring and monitoring iMg^2+^ is a more useful test to estimate patients’ Mg^2+^ status in order to prevent serious and potentially fatal complications of disease.

**Figure 2:**
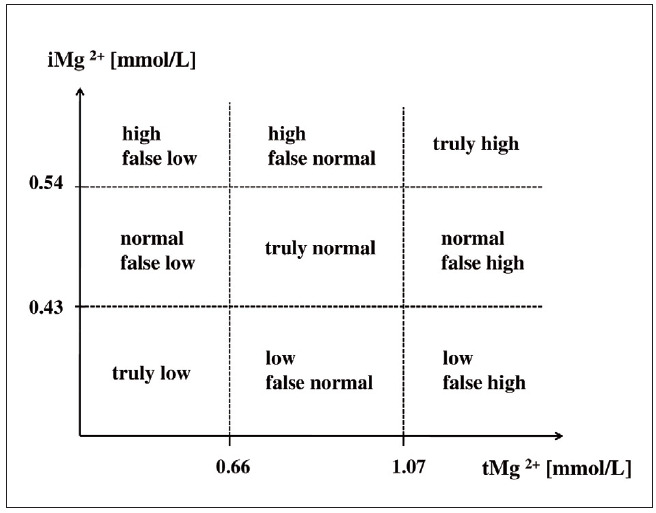
Magnesium status in the blood according to the results of measuring concentrations of total Mg^2+^ (tMg^2+^) and ionized Mg^2+^ (iMg^2+^) in the blood. When interpretating the findings, advantage should be given to the level of iMg^2+^ as the bioactive Mg^2+^ form. Magnesium status of an examenee can be high, normal or low. Total magnesaemia is correlated with ionized magnesaemia. Blood concentration of tMg^2+^ is shown as a function of iMg^2+^ blood concentration.

The interpretation of iMg^2+^ levels among clinicians is not entirely understood. To simplify the interpretation, the correlation between ionized magnesaemia and total magnesaemia can be presented graphically. Figure 2 illustrates how Mg^2+^ status of an examenee can be incorrectly interpreted, if it is judged only by measuring tMg^2+^, without assessing iMg^2+^ concentration in a given subject. Also mentioned are possible errors in concluding about Mg^2+^ status of the body based solely on the findings of tMg^2+^ levels in cases where levels of iMg^2+^ and tMg^2+^ do not show categorical agreement.

Magnesium status can be found to be low, normal or high, whereby the probability of making a wrong conclusion (false vs. real) is high if iMg^2+^ level is not included into the measurement as well, and interpretation is made only on the basis of the measured tMg^2+^. We can see how the finding of iMg^2+^ can actually better define what the actual Mg^2+^ status is. For example, low falsely normal Mg^2+^ status can be caused by alkalosis of the blood, a normal falsely low status – by hypoproteinaemia, a normal falsely high – by hyperproteinaemia, and a high falsely normal Mg^2+^ status – by acidosis. Situations are clear when levels of iMg^2+^ and tMg^2+^ are in accordance one to each other (either both normal or both changed in the same way), so that Mg^2+^ status in the blood is truly normal, or truly low, or truly high. However, in critical patients this is usually not the case, because the presence of the underlying disease and associated diseases and conditions (metabolic disorders, heart failure, circulatory insufficiency, kidney damage, neurological conditions, breathing disorders, etc.) usually leads to discrepancies, causing weak mutual correlation between ionized and total magnesiaemia in the critically ill. Ignoring these effects on the levels of the active form of Mg^2+^ in the blood, or neglecting to test for Mg^2+^ status at all, and especially including Mg^2+^ in the treatment protocol without considering iMg^2+^ first – all can lead to making a medical error that can further worsen the condition of these serious patients, due to the abovedescribed effects of Mg^2+^ on vital body functions.

Measurements show poor agreement between serum levels of tMg^2+^ and iMg^2+^ in adult patients admitted to ICUs [35]. In a significant number (up to 1/3) of patients in whom it was necessary to determine Mg^2+^ status, the changes in iMg^2+^ and tMg^2+^ levels were not in mutual concordance (possible finding of an increase in one concentration, with a simultaneous decrease of the other, or vice versa, or only one of the two values changed, while the other remains unchanged etc.). If only tMg^2+^ level is taken into account, a wrong conclusion about Mg^2+^ balance is often made, and sometimes even a wrong decision regarding treatment. This refers to both primary care patients and critically ill patients, adults and children. For example, ionized hypomagnesaemia together with normal total magnesaemia is present in many critically ill children, meaning that based only on routine tMg^2+^ testing they could not be recognized as being Mg^2+^-deficient [30].

Since circulating Mg^2+^ levels in the critically ill are often diminished predisposing them to many complications and even vital threat [36], the focus of this paper is on the group of critically ill patients in specific, due to a frequent presence of many associated metabolic and other disorders affecting iMg^2+^ levels. These include not only the disorders of the water-salt and acid-base balance and plasma proteins, but additionaly the changes in plasma concentrations of anionic ligands that bind Mg^2+^ ions into stable complexes. A significant increase in the level of lactates occurs during shock, citrates during transfusions of citrate-rich blood products, bicarbonates during their administration in the treatment of acidosis etc.). Finally, it is not a rare occasion for several such pathophysiological conditions to arise simultaneously in the same patient, making metabolic conditions even more complex. Under such conditions, the dynamic balance between different fractions of Mg^2+^ in the blood shifts almost instantly to one direction or another. Therefore, frequent and significant variations of iMg^2+^ with or without the accompanying changes in tMg^2+^ in critically ill patients require the inclusion of iMg^2+^ measurement during Mg^2+^ status assessment in the critically ill [34].

Literature results are however not fully consistent, as certain discrepancies in the conclusions of individual studies can be found regarding tMg^2+^ levels and iMg^2+^ levels as indicators of Mg^2+^ status of the body. One of the opposing opinions finds that tMg^2+^ and iMg^2+^ are closely correlated, but does not find a correlation between tMg^2+^ or iMg^2+^ and the severity of illness in ICU patients, whereby the authors conclude that iMg2+ does not need to be directly measured, but can rather be inferred from tMg^2+^ [37]. Other authors find an unpredictable relationshipbetween tMg^2+ ^and iMg^2+^ levels as measures of Mg^2+^ status, and an unclear relationship of Mg^2+^ level disorders and patient outcomes. They imply a limited current understanding of how to best define Mg^2+^ status and conclude that more systematic studies of Mg^2+^ in general critically ill patients are needed [38]. Until a unified opinion and an agreement on the value of iMg^2+^ as a parameter to assess Mg^2+^ status is met in the scientific community of basic researchers and clinical experts, the issue remains open for further studies, but unfortunately the endangered patients could also remain unattended.

### Chronic Mg^2+^ deficiency in critically ill patients

Why do we stress so much the issue of measuring iMg^2+^ for Mg^2+^ status and the issue of chronic Mg^2+^ deficiency in the critically ill patients? Reasons are many. Mostly because of the prevalence of Mg^2+^ deficiency and its impact on the outcome. Magnesium deficiency is a condition of depleted magnesium body stores as discovered by the finding of a positive magnesium retention in magnesium loading test. It is associated with increased disease risks [24]. Current estimates are that approximately 45–50% of Americans are magnesium deficient [39]. However, there is still no simple, fast and accurate laboratory test to determine the bodyʼs total Mg^2+^ status in humans. Thereby practical assessment of the true Mg^2+^ status is not an easy task. Initial stage of chronic Mg^2+^ deficiency is subtle and asymptomatic, i.e. clinically latent, tMg^2+^ levels are still normal, while Mg^2+^ depletion is present only on a cellular and tissue level [5]. This is the normomagnesemic type of Mg^2+^ deficiency, early in its natural course. Over time, Mg^2+^ deficiency slowly evolves (probably for many years) until it also appears in the blood. It remains subclinical until the level of hypomagnesaemia of 0.6 mmol/L or lower is reached [19].

Since Mg^2+^ is predominantly an intracellular cation, it is dificult to determine Mg^2+^ deficiency in the body, especially in its early stage of clinically unmanifested tissue Mg^2+^ deficit in the presence of normal ionized and total magnesaemia. Chronic latent Mg^2+^ deficiency in the body can only be detected by using special laboratory tests not routinely performed in clinical settings in general, nor in critical care settings, for example measuring the intracellular concentration of Mg^2+^. Malon et al. [40] suggest using the concentration of intracellular iMg^2+^ in erythrocytes as a parameter to establish reliable information on the functional Mg^2+^ status in critically ill postoperative patients. Measuring urinary excretion of Mg^2+^ before and after magnesium loading is another test that can be performed to determine Mg^2+^ retention as a sensitive biochemical marker of functional Mg^2+^ deficiency. Although intravenous (*i.v.*) magnesium loading test is demonstrated to be sensitive in detecting Mg^2+^ deficiency, using this assay to identify the condition in the critically ill has practical limitations, as it is time-consuming and contraindicated in cases with renal impairment. Thereby, many of these laboratory tests to assess Mg^2+^ status are usually not readily available to the practicing physician in ICU settings [3] [41].

At present, the most commonly used method to assess Mg^2+^ status is still measuring blood concentration of tMg^2+^. This, however, may not be the best method to evaluate Mg^2+^ status, as it does not reflect total body Mg^2+^ stores. Blood levels of tMg^2+^ depend on plasma proteins and may change without necessarily affecting the bioactive iMg^2+^ fraction. Furthermore, the correlation between tMg^2+^ in the blood and total body Mg^2+^ may be poor [42], since measurements can reveal normal tMg^2+^ despite the negative Mg^2+^ body stores [43]. For example, a large percentage of patients with arrhythmias have an intracellular Mg^2+^ deficiency, out of line with serum Mg^2+^ levels, which may help explain the rationale for the effectiveness of Mg^2+^ as an adjunct antiarrhythmic agent [44].

Since each of these alternative laboratory assessments has its own difficulties, complete procedure to assess total body magnesium actually has a greater theoretical and scientific research significance, due to a lot of practical limitations for their clinical implementation, especially under circumstances of intensive and emergency medical practice. At present there is no single simple, rapid and accurate laboratory test to indicate true Mg^2+^ body status. This, however, cannot diminish neither the prevalance, nor the clinical significance of a negative Mg^2+^ balance disorder in the critically ill. Regarding the ratio of iMg^2+^ : tMg^2+^ levels in the blood, a study concerned with determining the prevalence of Mg^2+^ deficiency in critically ill patients found the presence of hypomagnesaemia (low tMg^2+^) in 20–51% of cases and reduced iMg^2+^ levels in 14–66% of cases [45]. Thus, in a certain number of critically ill patients ionized hypomagnesaemia is present despite normal total magnesaemia, signifying the existence of a chronic latent Mg^2+^ deficiency in these patients, that had already depleted tissue Mg^2+^ stores. Thereby, it is important for the operating clinicians at ICU departments to always bare in mind the possibility of disturbances of Mg^2+^ balance and evaluate patients appropriately, if possible. In the settings of chronic latent Mg^2+^ deficiency already present in a patient, further Mg^2+^ depletion can easily develop under critical conditions, leading to a general state of hyperexcitability with systemic manifestations, including compromising patientʼs vital body functions [5].

On the mortality of critically ill patients due to severe dysmagnesaemia there are still no complete data. Several studies dealing with a practical question of why is it necessary to measure Mg^2+^ levels and assess Mg^2+^ status in critically ill patients, found multiple adverse effects of Mg^2+^ deficiency on vital body functions and patient survival. Hypomagnesaemia is associated with a statistically significantly longer stay in ICU, greater need for mechanical ventilator support, higher risk of sepsis and higher mortality in critically ill patients [41] [46]. More specifically, the development of ionized hypomagnesaemia in critical patients during their stay in ICU is associated with a worse prognosis due to longer hospitalization, higher prevalence of severe sepsis and septic shock, higher values of Sequential Organ Failure Assessment (SOFA) score and higher mortality rates. These findings point to a prognostic value of monitoring the iMg^2+^ levels [47]. On the other hand, hypermagnesaemia also represents a strong independent risk factor for lethal outcome in critically ill patients [48]. Hypermagnesaemia is a relatively rare electrolyte disbalance in clinical practice, but potentially life-threatening, as it is also associated with a longer stay in the ward, greater need for mechanical ventilation, and higher mortality [49]. We see that mortality prediction is actually associated with significant dysmagnesaemia of any type.

### Correction of disorders of Mg^2+^ status in critically ill patients

Despite literature findings of a frequently poor correlation of tMg^2+^ blood levels with total Mg^2+^ body stores, many hospitals face a major obstacle in that only few can measure iMg^2+^ levels and must rely on tMg^2+^ levels to guide replacement of Mg^2+^. This is however likely to be very inexact in the absence of iMg^2+^ measurements [50]. This review shows that clinicians should rely more on measuring iMg^2+^ levels, wherever possible, to assess Mg^2+^ status and determine whether supplemental Mg^2+^ is needed. Specific protocols for Mg^2+^ repletion need to be set according to indications. Before parenteral Mg^2+^ administration to a critically ill patient, it is also necessary to set a target therapeutic level for iMg^2+^ concentration, to reach it and maintain it while monitoring the patient. The dosage of a given Mg^2+^ preparation can be adjusted according to achieved iMg^2+^ levels as compared to the set goal.

Correcting Mg^2+^ status disorder guided by repeated measurements of iMg^2+^ helps to maintain sinus rhythm of the heart in a critically ill patient. In coronary unit patients it is necessary to maintain therapeutically higher target iMg^2+^ levels due to risk of fatal cardiac arrhythmias, with a continuous electrocardiogram (ECG) monitoring. For example, iMg^2+^ levels should be measured in patients with torsade de pointes type of polymorphic ventricular tachycardia, especially if it arises due to a drug induced QT interval prolongation [51]. Intravenous administration of MgSO_4_ can be used as an effective antiarrhythmic treatment in acute management of cardiac arrhythmias in critically ill patients with low serum iMg^2+^ [52]. Close intraoperative monitoring of iMg^2+^ andcorrection of its low levels should be required during cardiopulmonary surgery to reduce the risk of lifethreatening postoperative arrhythmias. A reduced incidence of ventricular tachycardia and a prolonged sinus rhythm duration can be achieved on the first postoperative day in cardiosurgical patients who receive Mg^2+^ supplementation administered parenteraly by giving MgSO_4_ if iMg^2+^ levels were below critical limit of < 0.5 mmol/L [53].

While the importance of monitoring and maintaining adequate Mg^2+^ status in cardiology and cardiosurgery is generally known, this is still not always the case in clinical practices treating electrolyte disorders of neurocritical patients. Neurointensive care as a relatively new medical field dealing with neurocritical care and treatment of patients with life-threatening neurological conditions (stroke, craniocerebral trauma, subarachnoid hemorrhage, status epilepticus, coma, brain edema, brain tumors, meningitis, encephalitis, etc.). However, an increasing preclinical and clinical knowledge regarding the pathophysiological role of Mg^2+^ deficiency in the brain is recently acknowledged in a number of neurological diseases: migraine, tension headache, Alzheimerʼs and Parkinsonʼs diseases, cerebral vasospasm, acute cerebral ischemia, traumatic brain injury, increased intracranial pressure etc. [11] [54]. Therefore, in neurocritical patients it is also necessary to check Mg^2+^ status for the presence of acute or chronic Mg^2+^ imbalances. Given that Mg^2+^ shows neuroprotective effects, good Mg^2+^ status can be considered to help improve the prognosis of these patients [55].

Both types of dysmagnesaemias and unstable Mg^2+^ levels in critically ill patients with different pathologies are associated with worse outcomes, than in critically ill patients with a preserved Mg^2+^ status [56]. Clinical studies and practice certify that correction of Mg^2+^ disorders has an important role in critical care, especially as they often arise in association with disbalances of other electrolytes (Na^+^, K^+^ and Ca^2+^ ions) in the same patient. Disorders of Mg^2+^ homeostasis in the blood are best detected by the procedure of measuring iMg^2+^ concentration which requires an analyzer with a Mg^2+^ ion-selective electrode, due to a need for rapid and reliable test results. Therefore, ICU departments should be equipped for an adequate Mg^2+^ status assessment, while physicians should be familiar with therapeutic protocols for Mg^2+^ *i.v.* recompense. The goal is to reach as close as possible to optimal Mg^2+^ status which may make an important contribution to primary and secondary disease prevention and to better outcome in critical patients.

It is indicated to give Mg^2+^ preparations for parenteral administration after establishing Mg^2+^ deficiency, usually as a slow *i.v.* injection or infusion of MgSO_4_ for at least 20–30 min for each 1 g of MgSO_4_. Dose can be repeted if necessary, which is usually the case in the depletion of total body stores of Mg^2+^. Slow injection / infusion time is important for adequate repletion, because Mg^2+^ can get slowly distributed into the tissues, but quickly eliminated by renal excretion [57]. After administration, it is necessary to monitor Mg^2+^ levels again, in order to correctly assess whether the compensation has been adequately carried out and the target blood levels of Mg^2+^ have been reached. Monitoring helps ensure that therapeutic range of Mg^2+^ levels (according to indication) is achieved, while preventing overdose to toxic Mg^2+^ levels considering the risk of iatrogenic hypermagnesaemia. Magnesium prophylaxis can be given for example to cardiac surgery patients to reduce the incidence of atrial fibrillation, one of the most common heart rhythm disorders in ICUs, associated with increased patient morbidity and mortality [58]. There is an ongoing randomized clinical trial that aims to determine the impact of perioperative continuous *i.v.* Mg^2+^ infusion given in order to achieve and maintain stable iMg^2+^ levels in the serum between 1.5 and 2.0 mmol/L, on the occurrence of postoperative atrial fibrillation related to cardiac surgery [59].

True Mg^2+^ status of the body is in fact very difficult to assess in the case of chronic latent Mg^2+^ deficiency, because it implies assessment of cellular and tissue Mg^2+^ stores. Namely, Mg^2+^ is the second most abundant intracellular cation. Therefore, Mg^2+^ blood levels may not reflect body Mg^2+^ status, since only less than 1% of total body Mg^2+^ is amenable to measurement in the extracellular fluid. Because of this, normal levels of Mg^2+^ in the blood cannot be used to exclude deficient body stores of Mg^2+^ [6]. By far the largest portion of total amount of Mg^2+^ in the body (about 99%) resides inside the cells. The remaining minor portion of total body Mg^2+^ present in the blood is what we deal with in clinical biochemistry when trying to assess and correct Mg^2+^ status. Therefore, determining iMg^2+^ levels certainly gives a more complete insight than knowing only tMg^2+^ levels of the subject in our attempts to be able to recognize actual Mg^2+^ deficiency. In fact, even those two findings are sometimes insufficiently informative. However, knowing that there is a dynamic balance between the intraand the extracellular iMg^2+^, we can value ionized magnesaemia as a fairly good and discriminating biomarker when estimating and monitoring Mg^2+^ status in a patient.

In any case, repeated measurements of iMg^2+^ after therapy aim to evaluate well Mg^2+^ status in the blood, after one part of the given dose of Mg^2+^ has certainly been distributed into the intracellular compartment, which we can judge only indirectly (especially in critical care patients, where treatment must be directed, fast and efficient). However, as it is technically still impossible to previously adequately clinically assess the exact degree of the present tissue Mg^2+^ deficiency in patientʼs body, we cannot either quite apropriately estimate the dosage of the total amount of Mg^2+^ required to adequately compensate for the total deficiency. This, together with the constant risk of Mg^2+^ toxicity due to overdose, justifies the need for repeated measurements of Mg^2+^ during its parenteral administration.

Initial hypermagnesaemia is asymptomatic (just like initial hypomagnesaemia), usually up to a level of about 2.1 mmol/L of tMg^2+^. Caution is therefore required when administering Mg^2+^. Together with the control measurements of Mg^2+^ concentrations, attention should also be given to physical findings. Hypermagnesaemia leads to muscle weakness (up to paralysis), weak or lost muscle reflexes, and finally cardiac arrhythmias and respiratory arrest. Magnesium is considered to be a drug of a wide therapeutic range, even when given parenterally, as it has low toxicity in people with preserved kidney function. The risk of hypermagnesaemia is high in patients with renal impairment due to an insufficient urinary Mg^2+^ excretion. During parenteral Mg^2+^ administration the patient may complain of weakness, nausea and hot flashes, sometimes even a significant drop in tension (especially if Mg^2+^ is injected too quickly), due to its vasodilative effect, which the patient should be warned about in advance.

Physiological decrease in iMg^2+^ levels occurs during the last trimester of pregnancy. MgSO_4_ is given in obstetrics for the prevention and control of convulsive seizures during pregnancy and labour to women with severe pregnancy-induced hypertension and preeclampsia/eclampsia syndrome [19]. Magnesium is often used in the ED and ICU to manage certain de novo supraventricular and ventricular tachyarrhythmias, and to treat hypomagnesemic tetany, pulmonary hypertension, attacks of migraine and tension headaches, severe asthma exacerbations, refractory hypokalaemia and hypocalcaemia, etc. For these given indications it is necessary to set a targettherapeutic concentration of iMg^2+^, and monitor for the achieved iMg^2+^ levels during parenteral administration. Do the benefitial therapeutic effects of Mg^2+^ in treating the majority of the above-mentioned difficult, serious and even life-threatening clinical conditions, actually speak in favor of the frequently present chronic latent Mg^2+^ deficiency participating in their etiopathogenesis and/or in favor of the effectiveness of Mg^2+^ as a simple and safe (sometimes alternative or supplementary) therapy for these conditions – remains yet to be determined. We are still lacking properly designed and conducted preclinical and clinical studies that could adequately evaluate this question.

## Discussion

Disorders of water and electrolyte balance are among the clinical problems most frequently encountered in ICU, contributing to the overall morbidity and mortality of critically ill patients. Magnesium is an electrolyte with very important pathophysiological perspectives for critically ill patients for several reasons. Derangements of Mg^2+^ balance are commonly found in these patients, which can result in poor clinical prognosis. Additionally, they are frequently exposed to a number of drugs that affect Mg^2+^ status in the body. Nevertheless, Mg^2+^ still does not manage to draw worthy clinical attention at ICU departments [60]. The aim of this paper was not to give a specific systematic review of all available literature on the present day state-of-the-art of measuring the concentration of iMg^2+^ in the blood of the critically ill patients, but rather to give an explanation on the clinical importance of iMg^2+^ measurement in these patients, as this type of analysis is not sufficiently applied in the clinical practice today. Under physiological conditions, the concentration of iMg^2+^ comprises for about 2/3 of total circulating Mg^2+^ concentration. It is the only bioactive form of Mg^2+^ and a more accurate indicator of the patientʼs Mg^2+^ status than tMg^2+^ concentration. Aditionally, it is possible that changes in iMg^2+^ levels do not correlate wellwith changes in tMg^2+^ in the same patient. Finally, iMg^2+^ is a valuable biomarker in critical care medicine considering the important role it has in proper function of excitable tissues and vital organs.

Patients with impaired Mg^2+^ status can rapidly be identified by point-of-care measurements of iMg^2+^ levels. Since ionized hypomagnesamia is shown to be a predictor of disease severity and mortality, the authors recommend monitoring iMg^2+^ levels in all patients admitted to ICU. It is necessary to correct the detected disorder of Mg^2+^ status in the overall care and management of these patients, as it significantly helps to stabilize vital body functions in the critically ill, and reduce their morbidity and mortality. Incorrect assessment of Mg^2+^ status based only on the measured levels of tMg^2+^ may lead to erroneous treatment decisions. Given that iMg^2+^ is the best currently clinically available indicator of total body Mg^2+^ status, its measuring can help determine correctly the need and protocol for Mg^2+^ repletion in the case that Mg^2+^ deficiency is found. Monitoring iMg^2+^ levels during and after therapy with parenteral Mg^2+^ could help improve patient outcome by enabling adequate control of the applied protocol for Mg^2+^ administration and replacement, while suppressing the risk of possible iatrogenic hypermagnesemia in the critically ill. Replanishing Mg^2+^ in deficient patients upon admission to ICU can help reduce morbidity and mortality of vitally endangered patients. New prospective clinical studies are needed to accurately evaluate this benefit for different subgroups of critically ill patients at multispeciality wards (cardiology, cardiosurgery, neurocritical, pulmology, pediatrics, etc.), in terms of better treatment and survival of these patients [23].

## Conclusion

The intention of this review is to give a thorough understanding of the value of measuring the concentration of iMg^2+^ fraction in the blood in critically ill patients, as a specific indicator of Mg^2+^ status of the body. Chronic Mg^2+^ deficiency likely to be present in the critically ill and even more likely to deepen during patient stay at intensive care, can significantly worsen the outcome. Severe Mg^2+^ deficiency induces the condition of generalized hyperexcitability, directly affecting vital body functions even up to the level of vital threat. Better awareness of the possible Mg^2+^ deficiency in the critically ill can result in a more adequate patient management and treatment. Its early detection based on low iMg^2+^ levels can guide Mg^2+^ repletion and help enable better prognosis for the critically ill. Concerning Mg^2+^ replacement in critically ill patients whose electrolyte analysis still shows Mg^2+^ levels (both tMg^2+^ and iMg^2+^) within the reference ranges, in order to replete Mg^2+^ tissue stores and prevent further Mg^2+^ depletion, there is currently very little evidence to support its benefit. This is not considered in clinical practice today, but should encourage future conduct of conclusive basic and translational research studies regarding the pathophysiology of chronic Mg^2+^ deficiency.

## Dodatak

### List of abbreviations

ED, emergency department; <br>ICU, intensive care unit;<br>tMg^2+^, total Mg^2+^;<br>iMg^2+^, ionized Mg^2+^;<br>pH, potentia *Hydrogenii *(Latin);<br>Pr, protein;<br>EDTA, ethylenediamine-tetraacetate;<br>*i.v.*, intravenous;<br>SOFA, sequential organ failure assessment;<br>ECG, electrocardiogram.

### Authors contributions

All authors have accepted responsibility for the entire content of this paper and approved the submission of the final manuscript.

### Acknowledgement

This work was supported by the Ministry of Education, Science and Technological Development of the Republic of Serbia, project number: 451-03-66/2024-03/200110.

### Conflict of interest statement

All the authors declare that they have no conflict of interest in this work.
